# Diagnostic difficulties and possibilities of NF1-like syndromes in childhood

**DOI:** 10.1186/s12887-021-02791-0

**Published:** 2021-07-29

**Authors:** Eva Pinti, Krisztina Nemeth, Krisztina Staub, Anna Lengyel, Gyorgy Fekete, Iren Haltrich

**Affiliations:** grid.11804.3c 0000 0001 0942 9821 II. Department of Pediatrics, Semmelweis University, Tuzolto utca 7-9, Budapest, 1094 Hungary

**Keywords:** Neurofibromatosis 1, Cafe-au-Lait spots, Neurofibroma, Child, National Institutes of Health (U.S.), Early diagnosis, Risk assessment, Genes, tumor suppressor, Oncogenes, Tumor predisposition, Neurofibromatosis type 1-like syndrome

## Abstract

**Background:**

Neurofibromatosis type 1 (NF1), which is caused by heterozygous inactivating pathogenic variants in the *NF1*, has poor phenotypic expressivity in the early years of life and there are numerous conditions, including many other tumor predisposition syndromes, that can mimic its appearance. These are collectively termed NF1-like syndromes and are also connected by their genetic background. Therefore, the NF1’s clinical diagnostic efficiency in childhood could be difficult and commonly should be completed with genetic testing.

**Methods:**

To estimate the number of syndromes/conditions that could mimic NF1, we compiled them through an extensive search of the scientific literature. To test the utility of NF1’s National Institutes of Health (NIH) clinical diagnostic criteria, which have been in use for a long time, we analyzed the data of a 40-member pediatric cohort with symptoms of the NF1-like syndromes’ overlapping phenotype and performed *NF1* genetic test, and established the average age when diagnostic suspicion arises. To facilitate timely identification, we compiled strongly suggestive phenotypic features and anamnestic data.

**Results:**

In our cohort the utility of NF1’s clinical diagnostic criteria were very limited (sensitivity: 80%, specificity: 30%). Only 53% of children with clinically diagnosed NF1 had a detectable *NF1* pathogenic variation, whereas 40% of patients without fulfilled clinical criteria tested positive. The average age at first genetic counseling was 9 years, and 40% of children were referred after at least one tumor had already been diagnosed. These results highlight the need to improve NF1-like syndromes’ diagnostic efficiency in childhood. We collected the most extensive spectrum of NF1-like syndromes to help the physicians in differential diagnosis. We recommend the detailed, non-invasive clinical evaluation of patients before referring them to a clinical geneticist.

**Conclusions:**

Early diagnosis of NF1-like syndromes can help to prevent severe complications by appropriate monitoring and management. We propose a potential screening, diagnostic and management strategy based on our findings and recent scientific knowledge.

**Supplementary Information:**

The online version contains supplementary material available at 10.1186/s12887-021-02791-0.

## Background

Neurofibromatosis type 1 (NF1), also known as von Recklinghausen’s disease, is caused by heterozygous inactivating pathogenic variations in the *NF1* gene. NF1 is the most frequent (prevalence 1–5/10000) neurocutaneous genetic disorder, which is characterized by an autosomal dominant (AD) inheritance pattern with complete penetrance, but very heterogeneous and age-related expressivity [1–3]. Its most distinctive features affect the skin, skeletal and nervous system. NF1 is associated with an increased risk for benign and malignant tumors and can be identified by genetic testing or clinical diagnostic criteria. The latter were assembled by The NIH consensus development conference in 1987 and are still in use [4]. The recommendation says that the diagnosis of NF1 should be established if the patient fulfills at least two criteria, or suspected if an individual meets only one of them:

- ≥6 café-au-lait macules (CALMs) (> 5 mm in greatest diameter in prepubertal individuals and > 15 mm in greatest diameter in postpubertal individuals),
- ≥2 neurofibromas of any type or ≥ 1 plexiform neurofibroma,
- freckling in the axillary or inguinal regions,
- optic pathway glioma (OPG),
- ≥2 Lisch nodules (iris hamartomas),
- a distinctive osseous lesion (sphenoid dysplasia or tibial pseudarthrosis),
- a first-degree relative (parent, sibling, offspring) with NF1 as defined by the above criteria.

The NIH criteria are both highly specific and highly sensitive in adults [5], but not in children. While almost 100% of patients meet the criteria by the age of 20 years, their sensitivity is 97% among eight-year-old children and only 30% by the age of 1 year in infants [6]. Furthermore, there are many other conditions which can mimic the NF1’s appearance and fulfill the abovementioned criteria. Among these NF1-like syndromes there are harmless conditions as well as severe disorders with potential life-threatening complications. They have different inheritance patterns and tumor predisposition effects, so they require diverse screening and therapeutic procedures. Therefore, it is important to identify and distinguish between them in time.

Several previous studies have dealt with the diagnostic challenge of NF1-like conditions [7–11]. Most of them established harmonious recommendations, but the number of open questions remains significant. Some highlighted the significance of certain clinical diagnostic aspects, while others proposed a molecular screening approach. Considering these facts, we attempted to collect all possible NF1-like conditions, evaluated their importance, surveyed the utility of NIH diagnostic criteria on a 40-member-large Hungarian cohort with NF1-like condition under 18 years of age, and analyzed their symptoms. To improve the deficiencies in the field of NF1-like syndromes’ in childhood we propose a possible method of examination, diagnosis, monitoring and management.

## Patients and methods

To estimate the number of syndromes/conditions that could mimic NF1, we compiled them through an extensive search of the scientific literature. To test the utility of NF1’s NIH clinical diagnostic criteria, which have been in use for a long time, we analyzed the data of a 40-member pediatric cohort with symptoms of the NF1-like syndromes’ overlapping phenotype and performed *NF1* genetic test.

### Search for NF1-like syndromes in online databases

With an extended search using the electronic databases PubMed, Online Mendelian Inheritance in Man (OMIM), Google Scholar, Microsoft Academic, Semantic Scholar and the Cochrane Library we attempted to collect all published NF1-like conditions. Every sign, symptom and term possibly associated to NF1 constituted the basis of our search.

### Recruitment and clinical characterization of pediatric patients in relation to NF1-like syndromes

A total of 40 patients (P1–40) under 18 years of age including 24 males (60%) and 16 females (40%) were recruited for this study in relation to the clinically suspected NF1-like syndrome from Semmelweis University’s II^nd^ Department of Pediatrics in Hungary. All children are attended by a licensed clinical geneticist and pediatrician, and undergo exhaustive physical examinations regularly. Age, family tree and patient history were registered during the first counseling and further tests (e.g. brain magnetic resonance imaging (MRI), cardiac ultrasound, endocrinological laboratory tests, etc.) were carried out when necessary. Regular extended medical check-ups include the following specialties/tests: ophthalmology (especially for the presence of Lisch nodules), otolaryngology (objective hearing test), dermatology (appearance and progression of skin lesions), neurology (status and systematic follow-up), abdominal ultrasound (intraabdominal tumors, renal artery stenosis, structure of great abdominal vessels and organs), cardiology (blood pressure measurements, echocardiography), laboratory tests (complete blood count, renal and hepatic function parameters, blood lipids and glucose, inflammatory parameters, iron status, 25-hydroxyvitamin D, calcium and phosphate level, microalbuminuria).

### Genetic tests

Every patient underwent exhaustive *NF1* variation testing: Giemsa-banding, fluorescent in situ hybridization (FISH), targeted multiplex ligation dependent probe amplification (MLPA), next-generation sequencing (NGS) and Sanger sequencing (Supplementary material 1).

Patients with signs suggestive of a condition other than NF1 were also tested for further gene variations (Supplementary material 2). Analyses of those genes were performed with the combination of FISH, targeted NGS and MLPA involving the entire coding region and flanking splice sites.

The de novo chromosome 4 inversion (46,XX,inv(4)(p13q13)) of a female patient was confirmed with FISH, but the breakpoints were not refined, because her family declined further genetic testing.

In every case all genetic tests were carried out on genomic deoxyribonucleic acid (DNA) isolated from peripheral blood sample.

Patients with contradictory genetic test results or variants of uncertain significance (VUS) were excluded from the cohort, thus only patients carrying known pathogenic variations are discussed in this paper. We excluded two patients from the cohort. One of them had an intronic *NF1* variation according to the first NGS, but it was not present with Sanger-sequencing or the repeated NGS from the same DNA sample. This patient was a 6.5-year-old male with 8 CALMs (each was over 5 mm in greatest diameter). Her adult father had 3 CALMs (each was over 15 mm in greatest diameter), but none of the parents had any *NF1* variation with NGS and Sanger sequencing. The other excluded female patient had a maternally inherited VUS. This index patient was 4 years old with 7 CALMs (each was over 5 mm in greatest diameter), but her mother did not have any symptoms.

### Patients’ classification to survey the utility of NIH criteria

To calculate sensitivity (SE) and specificity (SP) of the NIH diagnostic criteria in childhood, we created four categories of patients according to their clinical classification and *NF1* variation status: “clinically diagnosed and genetically confirmed NF1” patients have a pathogenic *NF1* variation and fulfill NIH criteria; “clinically not diagnosed but genetically detected NF1” children have a pathogenic *NF1* variation but do not fulfill NIH criteria; “clinically diagnosed but genetically not confirmed NF1” patients do not have a *NF1* pathogenic variation but fulfill NIH criteria; children with “clinically not diagnosed and genetically not detected NF1” have no pathogenic variation of *NF1* and do not fulfill NIH criteria.

SE and SP were calculated according to the following formulas:

$$ \mathrm{SE}\ \left(\%\right)=\frac{\mathrm{clinically}\ \mathrm{diagnosed}\ \mathrm{and}\ \mathrm{genetically}\ \mathrm{confirmed}\ \mathrm{NF}1\ \mathrm{patients}}{\mathrm{patients}\ \mathrm{with}\ \mathrm{pathogenic}\  NF1\ \mathrm{variation}}\ x\ 100. $$

$$ \mathrm{SP}\ \left(\%\right)=\frac{\mathrm{patients}\ \mathrm{without}\ \mathrm{clinically}\ \mathrm{diagnosed}\ \mathrm{and}\ \mathrm{genetically}\ \mathrm{detected}\ \mathrm{NF}1}{\mathrm{patients}\ \mathrm{without}\ \mathrm{pathogenic}\  NF1\ \mathrm{variation}}\ x\ 100 $$

## Results

### NF1-like is a wide, genotypic-phenotypic overlapping spectrum (Table 1)

According to the result of our search many different genetic conditions can mimic the NF1’s appearance, predominantly by their skin manifestations or tissue overgrowth. Certain disorders present real NF1-specific features, while others have similar changes/lesions of a different nature. Most of these entities have a distinct, well-described phenotype, however some might show a very confusing, atypical appearance. Albeit the group of NF1-like syndromes also includes harmless conditions, a great many are linked to cell proliferation/differentiation signaling pathways and are therefore associated with a higher risk for developing tumors. The largest subgroup consists of the RASopathies, which concern the Ras/mitogen-activated protein kinase (RAS/MAPK) network. Most disorders in this class are caused by activating pathogenic variations of different oncogenes that lead to increased tumor development risk in an AD inheritance pattern. Further classes strongly associated with higher tumor risk are the Phosphatase and tensin homolog (PTEN) hamartoma tumor syndromes, the Constitutional mismatch repair deficiency (CMMRD) syndromes, certain types of chromosomal aneuploidies or abnormalities, and the Multiple endocrine neoplasia (MEN) syndromes. PTEN hamartoma tumor syndromes also show an AD inheritance pattern and are characterized by many different localized tumors easily confused with neurofibromas. Fortunately, innocuous conditions can also be found among the NF1-like syndromes, causing only aesthetic lesions. Namely, genetic alterations involving the KIT ligand/c-KIT (KITLG/c-KIT) signaling pathway, which plays an important role in the regulation of skin pigment production, manifest only in pigment changes.

**Table 1:** NF1-like syndromes

DISEASE (OMIM/ORPHA ID [12, 13])	GENETIC BACKGROUND (INHERITANCE)	REFERENCES
RASopathies (RAS/MAPK signaling pathway)		
Neurofibromatoses:		[7, 9, 14, 15]
° NF1 (OMIM 162200): **• generalized/germline** **• segmental/mosaic/somatic** **• Watson sy.** **•** *AD multiple CALMs*	*NF1* (AD)	
° **NF2** (OMIM 101000)	*NF2* (AD)	
° **Schwannomatosis** (OMIM PS162091)	*LZTR1* (AD); *NF2* (AD); *SMARCB1* (AD)	
**Legius sy.** (OMIM 611431)	*SPRED1* (AD)	[7–9, 16–19]
**Noonan sy.** (OMIM PS163950)	50%: *PTPN11* (AD); 13%: *SOS1* (AD); 5%: *RAF1* (AD); 5%: *RIT1* (AD); < 5%: *KRAS* (AD); < 1%: *BRAF* (AD), *LZTR1* (AR/AD), *MRAS* (AD), *NRAS* (AD), *PPP1CB* (AD), *RRAS2* (AD), *SHOC2* (AD), *SOS2* (AD); 20%: unknown	[7–9, 20]
**LEOPARD sy.** (OMIM PS151100)	*BRAF* (AD); *MAP2K1* (AD); *PTPN11* (AD); *RAF1* (AD)	[7–9, 15, 21]
**Noonan sy. with loose anagen hair** (OMIM PS163950)	*SHOC2* (AD); *PPP1CB* (AD)	[22, 23]
**Cardiofaciocutaneous sy.** (OMIM PS115150)	*BRAF* (AD); *KRAS* (AD); *MAP2K1* (AD); *MAP2K2* (AD)	[8, 9, 21]
**Costello sy.** (OMIM 218040)	*HRAS* (AD)	[8, 21]
**CBL sy.** (OMIM 165360)	*CBL* (AD)	[24]
*Capillary malformation-arteriovenous malformation sy.* (OMIM PS608354)	*EPHB4* (AD); *RASA1* (AD)	[25]
KITLG/c-KIT signaling pathway		
*Piebaldism* (OMIM 172800)	*KIT* (AD); *SNAI2* (AD)	[8, 15, 26]
*Familial progressive hyperpigmentation sy.* (OMIM 614233)	*KITLG* (AD)	[8, 26]
*Familial progressive hyper- and hypopigmentation sy.* (OMIM 145250)		
PTEN hamartoma tumor syndromes		
**Proteus sy.** (OMIM 176920)	*AKT1* (unknown)	[15, 27]
**Cowden sy.** (OMIM PS158350)	*AKT1* (unknown); *KLLN* (unknown); *PIK3CA* (unknown); *PTEN* (AD); *SEC23B* (AD)	[7, 9, 15, 28–30]
**Peutz-Jeghers sy.** (OMIM 175200)	*STK11* (AD)	[15, 28]
Others		
**Ataxia-teleangiectasia** (OMIM 208900)	*ATM* (AR)	[7, 9, 31]
**Baraitser-Winter sy.** (OMIM PS243310)	*ACTB* (AD); *ACTG1* (AD)	[32–34]
**Bloom sy.** (OMIM 210900)	*RECQL3* (AR); *TOP3A* (AR)	[7, 9, 35]
**Carney complex** OMIM type 1:160980; type 2:605244)	type 1: *PRKAR1A* (AD); type 2: 2p16 (unknown)	[9, 36]
**CMMRD/DNA repair sys.** (OMIM 276300)	*MLH1* (AR); *MSH2* (AR); *MSH6* (AR); *PMS2* (AR)	[7, 9, 15, 28]
**Fanconi anaemia** (OMIM PS227650)	*BRCA1* (AR); *BRCA2* (AR); *BRIP1* (unknown); *ERCC4* (AR); *FANCA* (AR); *FANCB* (XLR); *FANCC* (AR); *FANCD2* (AR); *FANCE* (AR); *FANCF* (unknown); *FANCI* (AR); *MAD2L2* (AR); *PALB2* (unkown); *FANCL* (AR); *RAD51* (AD); *RAD51C* (AR); *RFWD3* (AR); *SLX4* (AR); *UBE2T* (AR); *XRCC2* (AR); *XRCC9* (unknown)	[7, 9, 37]
Fibromatoses /heterogenous group of multiple fibromas, most of them are benign/	unkown (possibly heterogenous, poligenic & multifactorial)	[15]
**Gaucher’s disease** (OMIM type 1: 230800; type 2: 230900; type 3: 231000; type 3C: 231005; perinatal lethal: 608013)	*GBA* (AR)	[38]
**Gorlin-Goltz sy.** (OMIM 109400)	*PTCH1* (AD); *PTCH2* (AD); *SUFU* (AD)	[9, 39]
**Jaffe-Campanacci sy.** (ORPHA 2029)	*NF1* (AD); but mostly unknown	[9]
Johanson-Blizzard sy. (OMIM 243800)	*UBR1* (AR)	[7, 40]
**Kabuki sy.** (OMIM PS147920)	*KDM6A* (XLD); *KMT2D* (AD)	[7, 41]
Klippel-Trenaunay-Weber sy. (OMIM 149000)	*PIK3CA* (unkown); but mostly unkown	[15, 42]
Lipomatoses /heterogenous group of multiple lipomas, they are nearly always benign/	unkown (possibly heterogenous, poligenic & multifactorial)	[15]
Marfan sy. (OMIM 154700)	*FBN1* (AD)	[43]
**Maternal UPD7p**	7p (AD)	[9]
McCune-Albright sy. (OMIM 174800)	*GNAS* (always de novo mosaicism)	[7, 9, 15, 44]
**MEN sys.:**		[7, 9, 15, 45]
° **MEN1 sy.** (OMIM 131100)	*MEN1* (AD)	
° **MEN2A sy.** (OMIM 171400)	*RET* (AD)	
° **MEN2B sy.** (OMIM 162300)		
Microcephalic osteodysplastic primordial dwarfism type II (OMIM 210720)	*PCNT* (AR)	[7, 46]
**Mosaic chromosomal tri−/monosomies (especially Turner sy.)**	heterogenous (usually de novo)	[7, 9, 47]
Multiple familial café-au-lait (ORPHA 2678)	unknown (unknown)	[7]
**Neurocutaneous melanocytosis** (ORPHA 2481)	unknown (unknown)	[15, 48]
**Nijmegen breakage sy.** (OMIM 251260)	*NBN* (AR)	[7, 9, 49]
**Proteus-like sy.** (ORPHA 2969)	50% *PTEN* (AD); 50% unknown (possibly PI3K pathway members’ mutations)	[50]
**Rapadilino sy.** (OMIM 266280)	*RECQL4* (AR)	[9]
**Ring 7/11/12/15/17/22 chromosome sys.**	mostly de novo, but sometimes inherited (AD)	[7, 9]
**Rothmund-Thomson sy.** (OMIM PS268400)	type 1: *ANAPC1* (AR); type 2: *RECQL4* (AR)	[9]
**Rubinstein-Taybi sy.** (OMIM PS180849)	*CREBBP* (AD); *EP300* (AD); del16p13.3 (unkown)	[7, 51]
**Silver-Russell sy.** (OMIM 180860)	35–50% ICR1 hypomethylation; 7–10% maternal UPD7; rarely: del/dup/t of chr7/11p15.5, *CDKN1C*, *IGF2*, *PLAG1*, *HMGA2;* 40% unkown (always AD)	[7, 9, 52]
**Tuberous sclerosis** (OMIM PS191100)	*IFNG* (AD); *TSC1* (AD); *TSC2* (AD)	[7, 9, 53]
**von Hippel-Lindau sy.** (OMIM 193300)	*CCND1* (AD); *VHL* (AD)	[54]

Disease names in bold: genetic conditions with increased tumor risk; disease names in italic: tumor predisposition with uncertain significance; *Abbreviations*: *AR* autosomal recessive, *CBL sy*. *CBL* gene mutation syndrome, *chr* chromosome, *del* deletion, *dup* duplication, *ICR1* imprinting control region 1, *OMIM/ORPHA ID* Online Mendelian Inheritance in Man/Orphanet Identifier, *p* short arm of a chromosome, *OMIM PS* Phenotypic Series in Online Mendelian Inheritance in Man (similar phenotypes with heterogeneous genetic background), *PI3K* phosphoinositide-3-kinase-protein kinase, *sy(s)*. syndrome(s), *t* translocation, *UPD* uniparental disomy

Many NF1-like syndromes show not only phenotypic but also genotypic overlap. Examples of this close relation are Noonan and Lentigines-Electrocardiographic conduction defects-Ocular hypertelorism-Pulmonary stenosis-Abnormalities of the genitalia-Retarded growth-Deafness (LEOPARD) syndromes (both can be caused by *Protein Tyrosine Phosphatase Non-Receptor Type 11* gene (*PTPN11*) pathogenic variation), or the *Phosphatidylinositol-4,5-Bisphosphate 3-Kinase Catalytic Subunit Alpha* gene (*PIK3CA*) pathogenic variation related Cowden and Klippel-Trenaunay-Weber syndromes. The NF1-like syndromes’ genetic background and diagnosis are further complicated by the fact that the disease-causing pathogenic variation is not always known (e.g. in 20% of Noonan syndromes [55]).

### Clinical characteristics and genetic results of the NF1-like pediatric cohort

#### Utility of the NIH criteria in childhood (Table 2)

Among 30 children with clinically diagnosed NF1 (P1–16 and P21–34) only 53% (16 patients) had a detectable pathogenic *NF1* variation. Ten individuals (P17–20 and P35–40) did not have clinically diagnosed NF1, but four of them (40%) had a detectable pathogenic *NF1* pathogenic variation (P17–20). In one further child (P35) additional genetic testing confirmed a *PTPN11* pathogenic variation causing Noonan syndrome. In the case of eight genetically NF1 negative patients with CALMs (P29–33 and P38–40) additional *Sprouty Related EVH1 Domain Containing 1* gene (*SPRED1*) analysis excluded the presence of Legius syndrome as well. Overall, the NIH criteria’s sensitivity was 80% (16/20 patients) and its specificity was 30% (6/20 patients) among the examined children.

**Table 2:** Cohort classification according to the NIH criteria and *NF1* pathogenic variation status

Clinically diagnosed and genetically confirmed NF1	Clinically not diagnosed but genetically detected NF1	Clinically diagnosed but genetically not confirmed NF1	Clinically not diagnosed and genetically not detected NF1
16 patients	4 patients	14 patients: • 3: Legius sy. • 2: NF2 • 1: MEN2B sy. • 1: LEOPARD sy. • 1: 46,XX,inv.(4)(p13q13)dn • 6: clinically diagnosed NF1	6 patients: • 1: Noonan sy. • 5: no definitive genetic diagnosis

There are four main groups according to the fulfillment of NIH criteria and detected *NF1* pathogenic variation

#### Components of the NIH criteria (Supplementary material 2)

Each NIH criterion represents the cardinal and/or most common symptoms of NF1. 40% of patients (8/20) with genetically confirmed NF1 had a de novo pathogenic variation. In one of the inherited cases (P19) the affected father did not meet the clinical criteria, he had only 1 CALM. Among children who had a clinical diagnosis of NF1, but tested negative for pathogenic *NF1* variations, there were three patients with Legius syndrome (P21–23), two with neurofibromatosis type 2 (NF2) (P24, 25), one with multiple endocrine neoplasia type 2B (MEN2B) (P26), one with LEOPARD syndrome (P27), and one with a 46,XX,inv(4)(p13q13) karyotype (P28). Six individuals without any detected *NF1* variation met the NIH criteria (P29–34) - most of them had CALMs and axillary/inguinal freckling -, but none of them had a detectable pathogenic *NF1* or *SPRED1* variation, or a characteristic phenotype suspicious of any other NF1-like conditions; they should probably have an undetected *NF1* variation. Two patients of them have clinically affected family members (P31: mother and brother, P32: mother). In these six cases (P29–34), we intend to perform *NF1* and *SPRED1* analysis of cultured melanocytes from biopsy of affected skin areas, and to carry out NF1-like gene panel test (including sequencing and copy number variation (CNV) analysis), and in the next step whole exome sequencing (WES) with CNV analysis or whole genome sequencing (WGS) from peripheral blood to exclude the presence of mosaicism or of other NF1-like conditions. Five further children fulfilled only one NIH criterion, and tested negative for an *NF1* pathogenic variation (P36–40).

#### Additional symptoms and anamnestic data (Supplementary material 2)

We collected every additional sign of patients possibly suggestive of NF1 or other NF1-like conditions, excluding those that are known frequent variations in the normal population. For example, 10% of healthy individuals have one or two CALMs, but the presence of three or more is rare (< 1%) and shows a relatively strong association with tumor predisposition syndromes [9]. Although isolated Lisch nodules can also occur in the normal population, they are more common in genetic abnormalities, such as an early sign of germline NF1, the only symptom of segmental NF1, or a rare feature of NF2 [56–58]. The same is true for the presence of single neurofibromas.

#### Occurrence of tumors (Table 3)

In total, 16 (40%) of 40 patients had 21 non-metastatic tumors. Among the different tumors, OPG occurred in the highest number (6/21, 29%): two in patients with verified *NF1*, one related to *NF2*, one to chromosome 4 abnormality, and two to *NF1* and *SPRED1* pathogenic variation double negative cases.

**Table 3:** Tumors of the patients

Patients (diagnosis)	Tumors
P2 (NF1^a^)	acoustic neuroma (unilateral), pituitary adenoma
P6 (NF1^a^)	ductal carcinoma of breast (unilateral)
P7 (NF1^a^)	OPG
P11 (NF1^a^)	OPG
P14 (NF1^a^)	acoustic neuroma (unilateral), pilocytic astrocytoma in the brain
P20 (clinically not diagnosed NF1, but detected by genetic test)	astrocytoma in the brain
P22 (Legius syndrome^a^)	epithelioma calcificans
P24 (NF2^a^)	OPG
P25 (NF2^a^)	meningioma, acoustic neuroma (bilateral)
P26 (MEN2B syndrome^a^)	medullary thyroid carcinoma
P27 (LEOPARD syndrome^a^)	rhabdomyosarcoma
P28 (46,XX,inv.(4)(p13q13) detected by genetic test)	OPG
P29 (clinically diagnosed NF1, but not detected by genetic test)	OPG
P34 (clinically diagnosed NF1, but not detected by genetic test)	OPG
P36 (no definitive genetic diagnosis)	ipsilateral tumors: thigh rhabdomyosarcoma, popliteal myopericytoma, symplastic hemangioma
P39 (no definitive genetic diagnoses)	forearm myxofibrosarcoma (unilateral)

^a^ Clinically diagnosed and confirmed by genetic test

30% (6/20) of children with pathogenic *NF1* variation and 50% (10/20) of patients without pathogenic *NF1* variation, as well as 27% (8/30) of NIH positive and 30% (3/10) of NIH negative individuals had a minimum of one neoplasm.

#### Sexes and ages of NF1-like pediatric patients

**Fig. 1:**
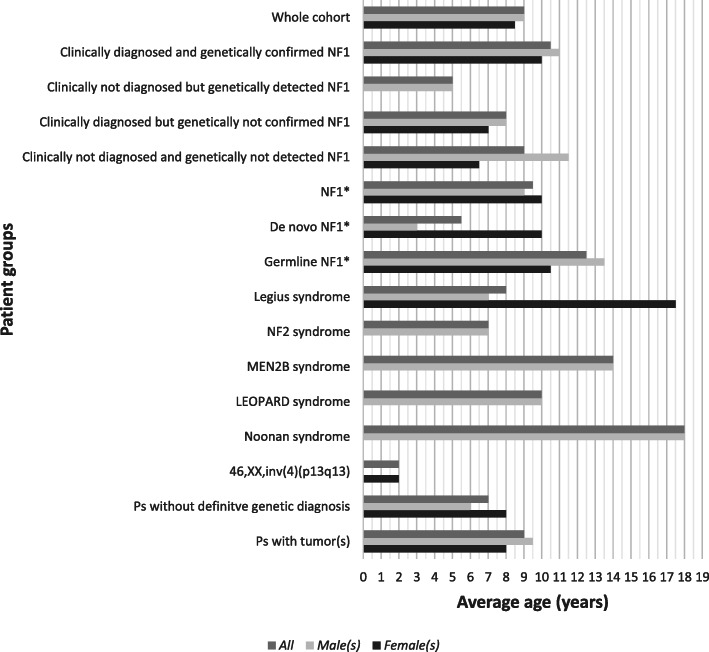
Average ages at the time of first genetic counseling and evaluation in relation to NF1-like syndromes. Each class is presented with three lines: mean age of the whole group, of males and of females. *: The group includes only patients having detected *NF1* pathogenic variant

Based on the ages of patients (Supplementary material 2) we calculated the average time of first genetic counseling in relation to NF1-like syndromes of different patient groups (Fig. 1). Each group is presented with three lines: mean age of the whole patient group, of males and of females.

Among the patients with pathogenic *NF1* variation 60% (12 children) were male and 40% (8 children) were female. Half of the 16 patients with clinically diagnosed and genetically confirmed NF1 were males and the other half were females. The group of the clinically not diagnosed but genetically detected NF1 patients included only boys (four patients). Five of the 14 clinically diagnosed but genetically not confirmed NF1 children were females and nine were males. The group of patients without clinically diagnosed and genetically detected NF1 included three females and three males. Half of the patients with pathogenic *NF1* variation had a de novo genetic alteration, five of them were male and three were female.

### Patient selection for genetic testing of NF1-like syndrome

To support the clinicians in the differential diagnosis, we collected the most extensive spectrum of possible NF1-like syndromes. According to the commonly overlapping phenotype, we recommend for clinicians a detailed, non-invasive clinical evaluation of patients before referring them to a clinical geneticist. Due to the age-related expressivity of most of the NF1-like syndromes, the regular follow-up is also recommended.

### Diagnostic and management strategy

Based on the symptoms we could choose the most efficient diagnostic method and the way of patient management. If phenotype and patient history is suspicious but not characteristic, we recommend targeted gene panels (including sequencing and CNV analysis of all NF1-like syndromes’ genes), and in case of a negative result, expansion to WES with CNV analysis or WGS as the most effective diagnostic approach. Here we should mention the diagnostic advantages and limitations of these extensive tests. The targeted gene panel requires less time, has lower cost, reaches higher coverage and sequencing depth than WES/WGS However, WES with CNV analysis or WGS could identify still unknown disease-causing genes, and they might also identify unexpected alterations (e.g. disease-causing repeat number in huntingtin gene) and/or many VUS. WES cannot identify CNVs of a certain size and localization.

In cases for which these tests are inconclusive, but clinical suspicion of NF1-like syndromes remains high, we advocate for ribonucleic acid (RNA)-based or epigenetic tests of suspicious genes, or to consider the possibility of a somatic or undetectable pathogenic variation. In instances of mosaicism the genetic tests might be negative due to the absence of peripheral blood lymphocytes’ involvement. To solve this problem, we should perform the tests on other types of tissues/cells possibly affected by the pathogenic variation (e.g. buccal epithelia). In cases when the genetic background is uncertain, we should consider the possibility of environmental effects or random, rare associations of normal, common phenotypic variants (harmless phenocopy). We should also emphasize that the detection rate of genetic tests - even in combination - do not reach 100%. Therefore, we should take into account the presence of an undetectable pathogenic variation in patients with a clinically diagnosed, but genetically not detected abnormality. Currently there are no methods that can help us clarify these eventualities.

For certain NF1-like syndromes disease-specific monitoring and management guidelines are available and are recommended for genetically confirmed NF1-like syndromes [15].

## Discussion

### NF1-like syndromes with unclarified tumor predisposing effect

As we have seen on Table 1 the relation between some conditions within this group and tumor predisposition remains unclarified. For instance, Legius syndrome, which is caused by the RAS/MAPK-member *SPRED1*’s pathogenic variation, behaves as an AD genetic disorder, even though the protein encoded by that gene acts as a tumor suppressor in a homo-, or heterodimer with its paralog Sprouty Related EVH1 Domain Containing 2 (SPRED2). Loss of function pathogenic variations of tumor suppressors are generally considered to have recessive expression patterns and are associated with increased risk of tumor development. However, extensive research over the last few years has elucidated that certain tumor suppressor genes might act as “double agents” with both oncogenic and tumor-suppressor functions. Therefore, in some cases heterozygous pathogenic variations can lead to manifest disease with a dominant inheritance pattern [59]. Legius syndrome is a demonstrative example. *SPRED1*’s loss of heterozygosity is thought to play a role in pediatric acute myeloblastic leukemia; furthermore, there is some evidence that Legius syndrome is possibly associated with an increased risk for kidney tumors and lung cancers [17–19]. Nonetheless, the definitive association between pathogenic *SPRED1* variations and tumor predisposition is complicated by the fact that Legius syndrome is very rare, therefore the amount of available evidence is currently insufficient. Whether there is a real increase of certain tumor types in Legius syndrome, or the reported cases represent random association awaits clarification in the future.

Until the significance of these diseases’ tumor predisposing effect remains unclarified, we recommend a regular (yearly) tumor screening/check-ups regarding to the tumors described in the literature. In our cohort, one patient with Legius syndrome (P22) had an epithelioma calcificans, which is a benign tumor and might be a random, independent lesion.

### Limitations of the NIH criteria in childhood and genetic testing

The relatively low, 80% sensitivity of the NIH criteria among the examined young patients can be the result of two factors. On the one hand, 46% of sporadic (de novo) cases with *NF1* pathogenic variation - which represent nearly half of all individuals with a pathogenic *NF1* variation - fail to meet the criteria by 1 year of age because of the absence of an affected first-degree relative. On the other hand, cardinal symptoms of NF1 usually appear in late childhood [11, 15]. Similar to the NIH criteria, genetic testing methods have limitations as well; it is known that there are rare pathogenic variations that are not detectable with genetic tests. In these cases, the NIH criteria could help us in recognition. Two (P31–32) of the six patients with fulfilled NIH criteria but without any detectable NF1 variation (P29–34) probably have a germline variation because of the clinically affected family members.

The 40% prevalence of de novo *NF1* pathogenic variations in our cohort approaches the 50% rate known from the scientific literature that is typical for monogenic disorders with an AD inheritance pattern and age-related expressivity. 75% (6/8) of presented patients with sporadic NF1 fulfilled the criteria, which is higher than the expected 46% [11]. However, DeBella and her colleagues’ results referred to children under 1 year of age, while our cohort included older patients.

Explaining the NIH criteria’s low specificity in the examined children is more complex. By reviewing the patients’ symptoms, we can see that several NF1-like conditions may also present with six or more CALMs, axillary and/or inguinal freckling, at least one clinically affected family member, and sometimes even neurofibromas and Lisch nodules. This shows that these criteria are the commonest, but not the most specific symptoms of NF1.

In the presented pediatric cohort NF1-like syndromes affected an approximately equal number of males and females, which was also true in the subgroup of patients with *NF1* pathogenic variation. The near equivalent efficacy of the NIH criteria in different sexes suggests that the prevalence and age-related appearance of the most cardinal NF1 symptoms are independent from gender. As there was a significant difference in the average age of patients with clinically diagnosed and genetically confirmed NF1 vs. children with clinically not diagnosed but genetically detected NF1 (10.5 vs. 5 years), the NIH criteria’s utility seems to be limited in early years of life. The difference between the mean ages of children with de novo and inherited *NF1* pathogenic variations (5 vs. 12 years) could be due to parental ascertainment bias: new and unknown symptoms in a family are more likely to cause concern than signs which are already present in family members. These latter familial, and usually painless symptoms are often perceived to be normal variants, and do not tend to urge parents to seek medical help.

The average age of patients having a tumor was 9 years, which highlights the need for NF1-like syndromes’ early diagnosis. The relatively high average age in MEN2B, LEOPARD and Noonan syndromes further represents the diagnostic challenge of these rare genetic disorders. In contrast, a chromosomal rearrangement can be discernible earlier due to frequent addition of congenital anomalies, which was illustrated by the case of a two-year-old patient with 46,XX,inv(4)(p13q13) karyotype.

As we have seen in a patient (P19) with inherited *NF1* pathogenic variation, the affected father did not meet the clinical diagnostic criteria. His case is instructive for many reasons. Firstly, we should note that there are rare cases in which the NIH criteria are not fulfilled despite entering adulthood. This phenomenon could be explained by NF1’s variable expressivity, the presence of mosaicism, or a type of pathogenic variation that causes only skin pigment lesions. Secondly, inheritance should not be based solely on clinical findings, genetic testing is always necessary to ascertain inheritance. Finally, the phenomenon of germline/gonadal mosaicism can explain uncertain heritability [60]: mosaicism of germinal cells can lead to apparently de novo pathogenic variations in the offspring, similar to some cases, which have been reported so far [61–64]. A further point of interest from the scientific literature is the observation of different *NF1*, or simultaneous *NF1* and *SPRED1* pathogenic variations within one family each [65], which can be attributed to *NF1*’s high variation rate and/or the phenomenon that the presence of one variation predisposes for another.

### Clinical relevance of additional symptoms and tumors in the NF1-like spectrum

NF1 is also associated with many characteristic features other than components of the NIH criteria (e.g. short stature, macrocephaly, unidentified bright objects (UBOs) on brain MRI, etc.), which can also present as first manifestations, thus young patients showing exclusively these symptoms do not receive the correct diagnosis, necessary tumor surveillance and therapy in time. Some of these features are very specific for NF1 but can be more difficult to diagnose (e.g. ascertaining the presence of UBOs requires an MRI, which necessitates anesthesia in young children). Naturally, this does not mean that we suggest performing routine brain scans in every suspected case indiscriminately, rather we propose that some of the additional symptoms should be considered when establishing clinical diagnostic criteria for NF1. UBOs are thought to have a weak relation with cognitive impairment, which is attributed to the possible presence of aberrant myelination or gliosis [15]. The tenuous link between UBOs and cognitive impairment is further weakened by our findings, i.e. that none of the patients with intellectual impairment had UBOs, and vice versa.

40% (16/40) of the cohort had at least one tumor and 10% (4/40) had two or more, which demonstrates the significant tumor predisposition effect of the entire NF1-spectrum. 30% (6/20) of the patients with genetically confirmed NF1 had minimum one tumor, 10% (2/20) of which were OPG, and this prevalence approaches the 15–20% cited in the scientific literature [66]. While children with NF1 have an increased risk for developing low-grade optic pathway and brainstem gliomas, the NF2 typically associates with low-grade tumors of cranial nerves (vestibular schwannomas), meninges (meningiomas) and spinal cord (ependymomas) [67]. This association is illustrated by two patients: patient (P20) with NF1 had an astrocytoma in the brain stem, and patient P25 with NF2 had a meningioma and bilateral acoustic neuromas. Although OPGs are thought to be usually specific to NF1, they were also found in other members of our cohort namely in one patient with NF2 (P24), one with the chromosome 4 rearrangement (P28), and two children without any detected pathogenic *NF1* or *SPRED1* variation (P29 and P34) (Table 3). These cases could be explained by the presence of mosaic *NF1* pathogenic variation, epigenetic alterations affecting *NF1*’s expression or the effect of its protein, as well as the association of this tumor type with other genetic diseases, or random, unfortunate association of sporadic OPG. Diagnosis of OPG with CT/MRI is possible in cases with characteristic imaging features, such as iso- or hyper-intense lesions on CT that usually enhance after contrast injection, or hypo- to iso-intense appearance on T1 MRI, and hyperintensity on T2 images. Biopsy of the suspicious lesion is offered to confirm the diagnosis only in patients with unusual clinical or imaging findings [68–72]. All OPG in our cohort showed characteristic appearance on MRI, therefore biopsy was not performed in our patients to avoid redundant, severe complications. The OPG of P24 with genetically detected pathogenic *NF2* variation was diagnosed neuroradiologically because of the OPG-characteristic appearance on MRI, therefore no biopsy from the tumor was performed.

After OPG, acoustic neuroma was the second most frequent tumor among the presented patients. At a young patient (P6) a unilateral ductal carcinoma of breast was detected. Higher incidence of breast cancer in NF1 is likewise known from the scientific literature, but its emergence in childhood is very rare. One patient with *NF1* pathogenic variation (P2) had pituitary adenoma that can cause further subsequent symptoms depending on its hormone production. Juvenile myelomonocytic leukemia (JMML) can also be a severe complication of NF1 [73], but did not occur in this cohort. The patient P36 had two plexiform neurofibromas and many other types of tumors in one area of the body, but there was no detectable *NF1* pathogenic variation in DNA isolated from peripheral blood sample, which was suggestive for segmental NF1. The abovementioned heterogeneity and development of tumors in NF1 can be attributed to the Knudson „two-hit” model. According to this concept, loss of heterozygosity is necessary for the development of benign tumors, especially neurofibromas, but not enough for the formation of malignancies. Evidence exists that further genetic aberration is required for malignant transformation in NF1 [74].

### Suggestions to improve the diagnostic efficiency of NF1-like syndromes in childhood

Considering the broad spectrum and diagnostic difficulties of overlapping diseases, we propose the comprehensive use of NF1-like conditions/syndromes, including NF1 as well as disorders with phenocopy of NF1. We emphasize the importance of an exhaustive clinical examination, family tree analysis and anamnesis, which essentially support the decisions regarding appropriate diagnostics and management. To guide physicians in the systematic assessment of a patient’s symptoms, and to assist in judging whether a child requires genetic evaluation, we provide a more detailed, non-invasive clinical evaluation of patients before referring them to a clinical geneticist.

### Management of patients with mosaic or clinically diagnosed but genetically undetectable NF1

As we cannot test all tissue types for *NF1* pathogenic variations, we do not know the extent of affected tissues. Therefore, patients with mosaic NF1 should be examined in the beginning and regularly followed-up until entering adulthood with the non-invasive tests also recommended for patients with non-mosaic NF1. Furthermore, patients with clinically diagnosed but genetically undetectable NF1 should be also regularly checked-up because of the high probability of cryptic pathogenic *NF1* variation and risk for NF1-related complications.

### Limitations and future perspectives

Unfortunately, it was not always possible to accurately register the time of the symptoms’ first appearance, due to the young age of the patients, parental uncertainty and/or lack of exact medical documentation. In most cases, the parents bring their children to the genetic counseling because of CALMs, which are commonly the first manifestation of NF1. Beside parents, the pediatricians also play an important role in recognition of NF1-like syndromes’ initial features. In Hungary the children are mandatorily checked-up at specific dates by well-trained home pediatricians. The precise registration of first presentation might be improved by careful patient and family education, which we plan to implement in the future.

The unusually high number of non NF1-related tumors could be the consequence of our clinic’s main profiles, one of which is pediatric tumors. Although the genetic division provides for patients with various genetic diseases (e.g. developmental delay, metabolic diseases, chromosomal abnormalities, congenital disorders, etc.), the number of children diagnosed with a tumor is significant. As the patients to this study were collected because of a wider spectrum of NF1-like syndromes and features, and consequently underwent NF1’s genetic testing, the cohort and our results could have a bias of more and mostly pediatric tumor related NF1-like syndromes. Although the number of patients managed by our clinic is limited, our results broaden the observations and support the knowledge about NF1 and other NF1-like conditions.

The diagnostic difficulties of NF1 have been discussed in the recent years and has led to an international consortium that revised the diagnostic criteria: the NIH criteria were retained with some changes and supplementary new elements (the recommendations are yet unpublished) [75–78]. According to this the following changes have been recommended:

- axillary or inguinal freckling should be bilateral;
- ophthalmic alterations could be not only ≥2 Lisch nodules, but also ≥2 choroidal abnormalities;
- distinctive osseus lesion could be not only sphenoid wing dysplasia, but also anterolateral bowing of tibia (tibial dysplasia), or pseudarthrosis of a long bone;
- the affected first degree relative with clinically diagnosed NF1 should be a parent;
- a new diagnostic element is the presence of an *NF1* heterozygous pathogenic variant.

According to the revised criteria, the classification of three patients’ cases in our cohort have been changed: P17, P18 and P20 fulfilled the new NF1 diagnostic criteria. Therefore, the sensitivity of the revised criteria was higher (93%) than according to the NIH criteria (80%), and the specificity did not decline (30%).

Our results emphasize the need for widespread use of the revised criteria, with the help of which the diagnostic effectivity of NF1 could be improved.

## Conclusions

We propose the consistent use of the term NF1-like syndromes for conditions showing NF1-like appearance, that may be useful in cases without a clarified genetic background, whereby we can shelter the patients from an unnecessary stigmatizing diagnosis and simultaneously emphasize the need of regular medical follow-up until adulthood. Due to the poor manifestation of NF1-like syndromes in childhood, we propose careful appraisal of additional signs and symptoms other than the NIH criteria and recommend yearly clinical reevaluation to detect newly emerging symptoms and to follow-up the patient’s development.

## Supplementary Information

**Additional file 1: Supplementary material 1.** *NF1* variation testing of the cohort. All 40 patients were tested for *NF1* pathogenic variation with combination of cyto- and molecular genetic methods.

**Additional file 2: Supplementary material 2.** Symptoms and data of the cohort. All patients were tested for NF1, but some also for other suspicious NF1-like genes based on the main symptoms. Fulfilled NIH criteria are highlighted in bold. *: The parent had clinical diagnosis confirmed by genetic test. Abbreviations: IUGR: intrauterine growth retardation.

## Data Availability

Not applicable.

## Abbreviations

*ACTB/G1*: *Actin Beta/Gamma 1* gene
AD: Autosomal dominant
*AKT1*: *AKT Serine/Threonine Kinase 1* gene
*ANAPC1*: *Anaphase Promoting Complex Subunit 1* gene
AR: Autosomal recessive
*ATM*: *ATM Serine/Threonine Kinase* gene
*BRAF*: *B-Raf Proto-Oncogene, Serine/Threonine Kinase* gene
*BRCA1/2*: *BRCA1/2 DNA Repair Associated* gene
*BRIP1*: *BRCA1 Interacting Protein C-Terminal Helicase 1* gene
CALM(s): Café-au-lait macule(s)
CBL sy.: *CBL* gene mutation syndrome
*CBL*: *Cbl Proto-Oncogene* gene
*CCND1*: *Cyclin D1* gene
*CDKN1C*: *Cyclin Dependent Kinase Inhibitor 1C* gene
chr: Chromosome
CMMRD: Constitutional mismatch repair deficiency
CNV(s): Copy number variation(s)
*CREBBP*: *CREB Binding Protein* gene
del: Deletion
DNA: Deoxyribonucleic acid
dup: Duplication
*EP300*: *E1A Binding Protein P300* gene
*EPHB4*: *EPH Receptor B4* gene
*ERCC4*: *ERCC Excision Repair 4, Endonuclease Catalytic Subunit* gene
*FANCA/B/C/D2/E/F/I/L*: *FA Complementation Group A/B/C/D2/E/F/I/L* gene
*FBN1*: *Fibrillin 1* gene
FISH: Fluorescent in situ hybridization
*GBA*: *Glucosylceramidase Beta* gene
*GNAS*: *GNAS Complex Locus* gene
*HMGA2*: *High Mobility Group AT-Hook 2* gene
*HRAS*: *HRas Proto-Oncogene, GTPase* gene
ICR1: Imprinting control region 1
*IFNG*: *Interferon Gamma* gene
*IGF2*: *Insulin Like Growth Factor 2* gene
*KDM6A*: *Lysine Demethylase 6A* gene
*KIT*: *KIT Proto-Oncogene, Receptor Tyrosine Kinase* gene
*KITLG*: *KIT Ligand* gene
KITLG/c-KIT: KIT ligand/c-KIT
*KLLN*: *Killin, P53 Regulated DNA Replication Inhibitor* gene
*KMT2D*: *Lysine Methyltransferase 2D* gene
*KRAS*: *KRAS Proto-Oncogene, GTPase* gene
LEOPARD: Lentigines-Electrocardiographic conduction defects-Ocular hypertelorism-Pulmonary stenosis-Abnormalities of the genitalia-Retarded growth-Deafness syndrome
*LZTR1*: *Leucine Zipper Like Transcription Regulator 1* gene
*MAD2L2*: *Mitotic Arrest Deficient 2 Like 2* gene
*MAP 2 K1/2*: *Mitogen-Activated Protein Kinase Kinase 1* gene
MEN: Multiple endocrine neoplasia
*MEN1*: *Menin 1* gene
*MLH1*: *MutL Homolog 1* gene
MLPA: Multiplex ligation dependent probe amplification
*MRAS*: *Muscle RAS Oncogene Homolog* gene
MRI: Magnetic resonance imaging
*MSH2/6*: *MutS Homolog 2/6* gene
*NBN*: *Nibrin* gene
NF1: Neurofibromatosis type 1
*NF1*: *Neurofibromin 1* gene
NF2: Neurofibromatosis type 2
*NF2*: *Neurofibromin 2* gene
NGS: Next-generation sequencing
NIH: National Institutes of Health (U.S.)
*NRAS*: *NRAS Proto-Oncogene, GTPase* gene
OMIM: Online Mendelian Inheritance in Man
OMIM ID: Online Mendelian Inheritance in Man Identifier
OMIM PS: Phenotypic Series in Online Mendelian Inheritance in Man (similar phenotypes with heterogeneous genetic background)
OPG: Optic pathway glioma
ORPHA ID: Orphanet: an online database of rare diseases and orphan drugs Identifier
p: Short arm of a chromosome
P: Patient
*PALB2*: *Partner And Localizer Of BRCA2* gene
*PCNT*: *Pericentrin* gene
PI3K: Phosphoinositide-3-kinase-protein kinase
*PIK3CA*: *Phosphatidylinositol-4,5-Bisphosphate 3-Kinase Catalytic Subunit Alpha* gene
*PLAG1*: *PLAG1 Zinc Finger* gene
*PMS2*: *PMS1 Homolog 2, Mismatch Repair System Component* gene
*PPP1CB*: *Protein Phosphatase 1 Catalytic Subunit Beta* gene
*PRKAR1A*: *Protein Kinase CAMP-Dependent Type I Regulatory Subunit Alpha* gene
*PTCH1/2*: *Patched 1/2* gene
PTEN: Phosphatase and tensin homolog
*PTEN*: *Phosphatase and tensin homolog* gene
*PTPN11*: *Protein Tyrosine Phosphatase Non-Receptor Type 11* gene
*RAD51*: *RAD51 Recombinase* gene
*RAD51C*: *RAD51 Paralog C* gene
*RAF1*: *Raf-1 Proto-Oncogene, Serine/Threonine Kinase* gene
RAS/MAPK: Ras/mitogen-activated protein kinase
*RASA1*: *RAS P21 Protein Activator 1* gene
*RECQL3/4*: *RecQ Like Helicase 3/4* gene
*RET*: *Ret Proto-Oncogene* gene
*RFWD3*: *Ring Finger And WD Repeat Domain 3* gene
*RIT1*: *Ras Like Without CAAX 1* gene
RNA: Ribonucleic acid
*RRAS2*: *RAS Related 2* gene
SE: Sensitivity
*SEC23B*: *SEC23 Homolog B, COPII Coat Complex Component* gene
*SHOC2*: *SHOC2 Leucine Rich Repeat Scaffold Protein* gene
*SLX4*: *SLX4 Structure-Specific Endonuclease Subunit* gene
*SMARCB1*: *SWI/SNF Related, Matrix Associated, Actin Dependent Regulator Of Chromatin, Subfamily B, Member 1* gene
*SNAI2*: *Snail Family Transcriptional Repressor 2* gene
*SOS1/2*: *SOS Ras/Rac Guanine Nucleotide Exchange Factor 1/2* gene
SP: Specificity
*SPRED1/2*: *Sprouty Related EVH1 Domain Containing 1/2* gene
SPRED2: Sprouty Related EVH1 Domain Containing 2 protein
*STK11*: *Serine/Threonine Kinase 11* gene
*SUFU*: *SUFU Negative Regulator Of Hedgehog Signaling* gene
sy(s).: Syndrome(s)
t: Translocation
*TOP3A*: *DNA Topoisomerase III Alpha* gene
*TSC1/2*: *TSC Complex Subunit 1/2* gene
*UBE2T*: *Ubiquitin Conjugating Enzyme E2 T* gene
UBO(s): Unidentified bright object(s)
*UBR1*: *Ubiquitin Protein Ligase E3 Component N-Recognin 1* gene
UPD: Uniparental disomy
*VHL*: *Von Hippel-Lindau Tumor Suppressor* gene
VUS: Variant of uncertain significance
WES: Whole exome sequencing
WGS: Whole genome sequencing
*XRCC2/9*: *X-Ray Repair Cross Complementing 2/9* gene
