# Supplementary material for: Diagnostic difficulties and possibilities of NF1-like syndromes in childhood
Source: BMC Pediatr. 2021 Jul 29;21:331. doi: 10.1186/s12887-021-02791-0 (PMC8320045; doi:10.1186/s12887-021-02791-0)
Supplement: Supplementary file 1 — Additional file 1: Supplementary material 1. NF1 variation testing of the cohort. All 40 patients were tested for NF1 pathogenic variation with combination of cyto- and molecular genetic methods. [file 12887_2021_2791_MOESM1_ESM.docx]

**Supplementary material 1.**

**Title:** *NF1* variation testing of the cohort

**Legend:** All 40 patients were tested for *NF1* pathogenic variation with combination of cyto- and molecular genetic methods.

NF1 variations were screened at all patients by:

o 1^st^ Step: Conventional Giemsa-banding to detect any chromosomal rearrangement, which was performed on 15 Giemsa-stained metaphases from standard 72-hour peripheral blood lymphocyte cultures.

o 2^nd^ Step: Fluorescent in situ hybridization of 200 interphase cells from peripheral blood with *NF1* specific 17q11 probe (Kreatech, Amsterdam, Netherlands) was performed to exclude whole *NF1* deletion and its mosaicism.

o 3^rd^ Step: *NF1* variation detection with MLPA as Polgar and its colleagues described previously in 2011 (Polgár N, Komlósi K, Hadzsiev K, Illés T, Melegh B. [Molecular genetic diagnosis of neurofibromatosis type I]. Orv Hetil. 2011; 152(11): 415-419. <https://doi.org/10.1556/OH.2011.29059>).

o 4^th^ Step: *NF1* variation detection with NGS and validation with Sanger sequencing.

- Preparation of genomic DNA: DNA was prepared and amplified from peripheral blood samples in a total volume of 25 µl according to the protocol of the High Pure PCR Template Purification kit (Roche Diagnostics GmbH, Mannheim, Germany) or Bioline Immomix (Roche Diagnostics GmbH, Mannheim, Germany). The PCR reaction began with an initial denaturation step at 95°C for 7 min and was followed by 35 cycles of 94°C for 1 min, 65°C for 45 s and 72°C for 30 s, together with a final extension step at 72°C for 10 min.
- NGS: analysis was performed on Illumina HiSeq (2500/4000) instrument using the SureSelect Human All Exon V6 kit for library preparation.
  - Coverage: 99.03% (all exons and 100 bp introns)
  - Average Depth: 74x
- Sanger sequencing: was performed on an ABIPRISM 310 genetic analyser using BigDye terminator cycle sequencing kit v.3.1 (Applied Biosystems, Foster City, CA).
- The sequences were compared with: the revised Cambridge reference sequence (https://www.ncbi.nlm.nih.gov/nuccore/213385299; NCBI Reference Sequence: NG_009018.1).
