# Supplementary material for: Diagnostic difficulties and possibilities of NF1-like syndromes in childhood
Source: BMC Pediatr. 2021 Jul 29;21:331. doi: 10.1186/s12887-021-02791-0 (PMC8320045; doi:10.1186/s12887-021-02791-0)
Supplement: Supplementary file 2 — Additional file 2: Supplementary material 2. Symptoms and data of the cohort. All patients were tested for NF1, but some also for other suspicious NF1-like genes based on the main symptoms. Fulfilled NIH criteria are highlighted in bold. *: The parent had clinical diagnosis confirmed by genetic test. Abbreviations: IUGR: intrauterine growth retardation. [file 12887_2021_2791_MOESM2_ESM.docx]

**Supplementary material 2.**

**Title:** Symptoms and data of the cohort

**Legend:** All patients were tested for NF1, but some also for other suspicious NF1-like genes based on the main symptoms. Fulfilled NIH criteria are highlighted in bold. *: The parent had clinical diagnosis confirmed by genetic test. Abbreviations: IUGR: intrauterine growth retardation.

| PATIENTS | OTHER TESTED GENE(S) | DIAGNOSIS | SEX | FIRST GENETIC EVALUATION | | OTHER ANAMNESTIC DATA AND SYMPTOMS | AFFECTED PARENT | INHERITANCE |
| --- | --- | --- | --- | --- | --- | --- | --- | --- |
|  |  |  |  | AGE IN MONTH(S) | REASON FOR REFERRAL |  |  |  |
| ***CLINICALLY DIAGNOSED NF1 CONFIRMED BY GENETIC TEST*** | | | | | | | | |
| P1 | no | NF1 | M | 48 | ≥6 CALMs, bilateral axillary freckling | - | no | *de novo* |
| P2 | *NF2:* negative | NF1 | F | 7 | ≥6 CALMs, bilateral axillary freckling | bone dysplasia, osteopenia, precocious puberty, unilateral acoustic neuroma, pituitary adenoma | no | *de novo* |
| P3 | no | NF1 | M | 6 | ≥6 CALMs, bone dysplasia, anterolateral tibial bowing | - | no | *de novo* |
| P4 | no | NF1 | F | 173 | ≥6 CALMs, bilateral axillary and inguinal freckling, | scoliosis, gait abnormality, nystagmus | yes* | germline |
| P5 | no | NF1 | F | 212 | ≥6 CALMs, ≥2 neurofibromas | - | yes* | germline |
| P6 | no | NF1 | F | 209 | ≥6 CALMs, bilateral axillary freckling, ≥2 neurofibromas | scoliosis, ductal carcinoma of breast (unilateral) | no | *de novo* |
| P7 | no | NF1 | F | 64 | ≥6 CALMs, bilateral inguinal freckling, ≥2 Lisch nodules, intellectual disability, IUGR | renal artery stenosis, short stature, OPG | yes* | germline |
| P8 | no | NF1 | F | 19 | ≥6 CALMs, bilateral axillary freckling, ≥2 neurofibromas, relative macrocephaly, intellectual disability | ≥2 Lisch nodules, scoliosis | yes* | germline |
| P9 | no | NF1 | M | 206 | ≥6 CALMs, ≥2 neurofibromas | bilateral axillary freckling | yes* | germline |
| P10 | no | NF1 | M | 60 | ≥6 CALMs, bilateral axillary freckling | ≥2 neurofibromas | yes* | germline |
| P11 | no | NF1 | F | 132 | ≥6 CALMs, tibial non-ossifying fibroma, | OPG | no | *de novo* |
| P12 | no | NF1 | M | 94 | ≥6 CALMs, bone dysplasia | exophthalmos, cranial asymmetry | no | *de novo* |
| P13 | no | NF1 | M | 215 | ≥6 CALMs, bilateral axillary and inguinal freckling, ≥2 neurofibromas | UBOs | yes* | germline |
| P14 | *NF2:* negative | NF1 | M | 207 | ≥6 CALMs, bilateral axillary freckling, ≥2 neurofibromas, epilepsy, unilateral acoustic neuroma, pilocytic astrocytoma in the brain | - | yes* | germline |
| P15 | no | NF1 | F | 169 | ≥6 CALMs, bilateral inguinal freckling | ≥2 neurofibromas | yes* | germline |
| P16 | no | NF1 | M | 213 | ≥6 CALMs, bilateral axillary and inguinal freckling, ≥2 neurofibromas | - | yes* | germline |
| ***CLINICALLY NOT DIAGNOSED NF1 BUT DETECTED BY GENETIC TEST*** | | | | | | | | |
| P17 | *SPRED1:* negative | NF1 | M | 12 | ≥6 CALMs | ≥6 CALMs, talipes equinovarus | no | *de novo* |
| P18 | no | NF1 | M | 208 | affected parent (family screening) | asymptomatic | yes* | germline |
| P19 | *SPRED1:* negative | NF1 | M | 21 | 2 CALMs, cryptorchidism | 2 CALMS, cryptorchidism | yes (detected by gene test, but the father had only 1 CALM) | germline |
| P20 | *SPRED1:* negative | NF1 | M | 3 | ≥6 CALMs | ≥6 CALMs, UBOs, epilepsy, astrocytoma in the brain | no | *de novo* |
| ***FULFILLED NIH CRITERIA WITHOUT DETECTED NF1 PATHOGENIC VARIATION*** | | | | | | | | |
| P21 | *SPRED1:* pathogenic variant | Legius syndrome | F | 81 | ≥6 CALMs, and mother with NF1 symptoms (≥6 CALMs, bilateral axillary and inguinal freckling) | - | yes* (the mother had ≥6 CALMs, bilateral axillary and inguinal freckling) | germline |
| P22 | *SPRED1:* pathogenic variant | Legius syndrome | M | 202 | ≥6 CALMs, bilateral axillary freckling, | epithelioma calcificans | no | *de novo* |
| P23 | *SPRED1:* pathogenic variant | Legius syndrome | M | 8 | ≥6 CALMs, bilateral inguinal freckling | - | yes* (the father had ≥6 CALMs, bilateral inguinal freckling) | germline |
| P24 | *NF2:* pathogenic variant | NF2 | M | 65 | ≥6 CALMs, ≥2 neurofibroma, | OPG | no | *de novo* |
| P25 | *NF2:* pathogenic variant | NF2 | M | 107 | ≥6 CALMs, ≥2 Lisch nodules | meningioma, bilateral acoustic neuroma | no | *de novo* |
| P26 | *RET:* pathogenic variant | MEN2B syndrome | M | 167 | ≥6 CALMs, bilateral axillary and inguinal freckling, Marfanoid habitus, generalized muscle weakness, | multiple mucosal neuromas, medullary thyroid carcinoma | no | *de novo* |
| P27 | *NF2, BRAF, MAP2K1,* and *RAF1:* negative, *PTPN11:* pathogenic variant | LEOPARD syndrome | M | 130 | ≥6 CALMs, bilateral inguinal freckling, developmental delay, bilateral ptosis, bilateral sensorineural hearing impairment, craniofacial dysmorphism, rhabdomyosarcoma | pulmonary valve stenosis | no | *de novo* |
| P28 | no | 46,XX,inv(4)(p13q13) | F | 22 | ≥6 CALMs, bilateral axillary freckling, sphenoid dysplasia, relative macrocephaly, scoliosis, intellectual disability, epilepsy, IUGR, oligohydramnios | mitral insufficiency, mitral prolapse, OPG | no | *de novo* |
| P29 | *PTPN11:* negative | NF1 | F | 106 | ≥6 CALMs, ≥2 Lisch nodules, osteopenia, learning difficulties, behavioral disorders, scapular asymmetry, pes planovalgus, lumbal spina bifida, permanently decreased immunoglobulin A and G levels, | ≥2 neurofibromas, OPG | no | *de novo* |
| P30 | *SPRED1:* negative | NF1 | F | 10 | ≥6 CALMs, bilateral axillary freckling | - | no | *de novo* |
| P31 | *SPRED1:* negative | NF1 | M | 121 | ≥6 CALMs, bilateral axillary and inguinal freckling, hypertension | - | yes (the mother had ≥6 CALMs and bilateral axillary freckling) | germline |
| P32 | *SPRED1:* negative | NF1 | F | 211 | ≥6 CALMs, bilateral inguinal freckling, ≥2 neurofibromas, | UBOs | yes (the mother had ≥6 CALMs and bilateral axillary and inguinal freckling) | germline |
| P33 | *SPRED1:* negative | NF1 | M | 42 | ≥6 CALMs, bilateral axillary and inguinal freckling, 1 umbilical plexiform neurofibroma | - | no | *de novo* |
| P34 | *MEN1* and *RET:* negative | NF1 | M | 15 | ≥2 Lisch nodules, bilateral anterolateral tibial bowing, precocious puberty, Marfanoid habitus, OPG | - | no | *de novo* |
| ***CLINICALLY NOT DIAGNOSED NF1 AND NOT DETECTED BY GENETIC TEST*** | | | | | | | | |
| P35 | *PTPN11:* pathogenic variant | Noonan syndrome | M | 215 | scoliosis and pulmonary valve stenosis (which occur also in NF1) | intellectual disability, craniofacial dysmorphism | no | *de novo* |
| P36 | no | no definitive genetic diagnosis | F | 209 | 2 histologically confirmed plexiform neurofibromas | ipsilateral tumors: thigh rhabdomyosarcoma, popliteal myopericytoma, symplastic hemangioma | no | *de novo* |
| P37 | no | no definitive genetic diagnosis | M | 105 | multiple bone diseases (which occur in NF1: unilateral tibial cyst, pseudoarthrosis, non-ossifying fibroma) | - | no | *de novo* |
| P38 | *SPRED1* and *NF2:* negative | no definitive genetic diagnosis | F | 17 | ≥6 CALMs and positive family history for juvenile brain tumor | - | no (but the paternal grandfather had a brain tumor < 40 years of age) | *de novo* or germline with incomplete penetrance and variable expressivity |
| P39 | *SPRED1:* negative | no definitive genetic diagnosis | F | 4 | ≥6 CALMs also at her mother | unilateral forearm myxofibrosarcoma | no (but the mother also had ≥6 CALMs) | germline |
| P40 | *SPRED1:* negative | no definitive genetic diagnosis | M | 91 | ≥6 CALMs and positive family history for ≥2 neurofibromas | hypertension | no (but the maternal grandmother had ≥2 histologically confirmed neurofibromas) | *de novo* or germline with incomplete penetrance and variable expressivity |
